# Cancer care and public health policy evaluations in France: Usefulness of the national cancer cohort

**DOI:** 10.1371/journal.pone.0206448

**Published:** 2018-10-31

**Authors:** Philippe Jean Bousquet, Delphine Lefeuvre, Philippe Tuppin, Marc Karim BenDiane, Mathieu Rocchi, Elsa Bouée-Benhamiche, Jérôme Viguier, Christine Le Bihan-Benjamin

**Affiliations:** 1 Survey, Monitoring and Assessment Department, Public Health and Healthcare Division, Institut National du Cancer (French National Cancer Institute—INCa), Boulogne Billancourt, France; 2 Economy and social science, health care systems and societies, Faculté de médecine, Marseille, France; 3 Caisse nationale d'assurance maladie des travailleurs salariés, département des études sur les pathologies et les patients (DEPP), Paris, France; 4 Public Health and Healthcare Division, Institut National du Cancer (French National Cancer Institute—INCa), Boulogne Billancourt, France; University of Toledo, UNITED STATES

## Abstract

**Background:**

In the context of the national Cancer Plans of France that have changed the healthcare landscape, it has become necessary to better document and assess the related actions, and to promote research and understanding. The national cancer cohort, an exhaustive population-based cohort, was set up on the basis of the National Health Data System (SNDS) by the French National Cancer Institute.

**Objectives:**

The aim is to describe the French national cancer cohort.

**Methods:**

All people living in France (67 million population) with universal insurance coverage and diagnosed, treated or followed up for a cancer, such as survivors, are included and will be followed up for 25 years. It contains all healthcare consumptions and reimbursements (*i*.*e*. hospitalization, outpatient care, medication…) since 2010. Every year, around 650 000 new cases are included.

**Results:**

From 2010 to 2015, 6.2 million subjects have been included. Most subjects were entered in 2010, in 2015 it concerned 0.6 million. In 2015, the median age was 65 [54–76]; 51% were women. The primary cancer organ could be attributed with certitude to 87% of the people. The most frequent locations were skin (16%), breast (15%), prostate (12%), colon-rectum (11%) and lung (9%). In 2015, 40% of included subjects underwent surgery for cancer, 16% chemotherapy at hospital and 11% at least one session of radiotherapy.

**Conclusion:**

Based on SNDS, the cancer cohort has been designed to study cancer care use in the short-, medium- and long-term, and evaluate healthcare and public health policies.

## Background

Since 2003, three national Cancer Plans of France (a set of actions conducted by the Ministry of Health, which aims to bring together forces available within a country to fight against cancer) [1, 2, 3] have changed the healthcare landscape by setting up organised national screening (breast and colon cancer and recently cervical cancer), promoting prevention either in the general population, workers and targeted populations such as teenagers [4], reinforcing care pathways (hospital authorisation for cancer care, multidisciplinary staff, standardised documents for better coordination, specific care pathways for complex or rare cancers, etc.), and improving life during and post-cancer (focusing on both quality-of-life and employment, and reinforcing supportive care). These actions have extensively modified the organisation of healthcare for cancer and had a marked impact on healthcare professionals. Furthermore, the number of new cancer cases is on the rise, in line with the growth and aging of the French population. Simultaneously, overall and net survival rates are improving and leading to better life expectancy after cancer diagnosis [5, 6]. This is mainly due to medical developments and therapeutic innovations such as targeted therapies or immunotherapies, alongside public health policies aimed at preventing and detecting early cancer, and improving care for subjects with cancer.

Although cancer could be considered to be a common disease for a population of around 67 million inhabitants, with 400,000 new cases (invasive cancers) in 2017 in France (214,000 men and 186,000 women), and around 3 million persons treated in 2014, it represents a wide array of diseases [7, 8]. The four main sites represent only 50% of incident cases in France: breast cancers among women (59,000 incident cases), prostate cancers (48,000), lung cancers (49,000 cases) and colorectal cancers (45,000 cases) in both sexes [7]. Nevertheless, some cancers of interest, such as thyroid cancers (fewer than 10,600 cases) or testicular cancers (2,300 men), are less common and require national data to be studied. Moreover, with 643 thousand square kilometres and 5 overseas departments, studies on geographic disparities or inequities are mandatory. Others focusing on specific populations such as the elderly or children and teenagers (1700 new cases diagnosed every year in children and adolescents under 15 years), or the medium- and long-term, and unexpected side-effects especially for innovative therapies (which may be given to hundreds of patients *i*.*e*. fewer than 0.1% of the incident cases of cancer) have to be considered. Therefore, it becomes necessary to better document and assess Cancer plan actions, and also to promote research and understanding. Currently in France, cancer epidemiological data are mainly available through regional cancer registries with national extrapolation and national mortality statistics based on death certificates [9, 5, 7].

France provides universal medical coverage to all citizens. In 1999, French legislators asked health insurance funds to develop a National Health Insurance Information System in order to determine more accurately and evaluate the healthcare use and expenditure of beneficiaries. In 2016, the cornerstone of the National Health Data System *(Système National des Données de Santé*, SNDS) includes individual information on the sociodemographic, diagnostics and healthcare use of beneficiaries, such as all hospital care and dispensed medicine reimbursements [10].

According to SNDS, in 2014, 3 million people (4.4%) in France had a managed cancer (men 1.44 million; women 1.56 million). National cancer-related expenditure was 16 billion euros, among them 6 billion for primary health care (drugs 2.7 billion) and 9 billion for hospitals (drugs 1.6 billion) [8].

In this context, the French cancer cohort, a population-based cohort, was set up on the basis of SNDS by French National Cancer Institute (*Institut National du cancer*, *INCa*). The main objective of the cancer cohort is to provide a robust and validated database for creating dashboards, paying particular attention to complications, side effects, morbidity and excess mortality by cancer subtype or post-treatment and for supporting research. This should allow in particular 1) to have simple, validated and reproducible activity and expenditure indicators and to allow a regular monitoring of consumption, both in volume and in terms of expenditure, inherent to diagnosis, health care and actions; 2) to have simple, validated and reproducible quality and safety of care indicators including process and outcome indicators; 3) to observe the evolutions of these consumptions or indicators; 4) to follow the geographical distribution of specific tracer acts of certain diagnostic, therapeutic, health care or action modalities including prevention; 5) to study the care pathways of people with cancer or at high risk of cancer.

The aim of this paper is to describe the French cancer cohort: data sources, inclusion criteria, data description, access, and first planned analyses.

## Methods

### Population and follow-up

The cancer cohort was intended to be exhaustive, including all citizens living in France with universal insurance coverage (including immigrants). Consequently, all subjects diagnosed, treated or followed up for a cancer and survivors are included. After inclusion, a subject will be followed up for 25 years (data collection being updated yearly). The start date is 1 January 2010. As the cohort is prospective, each year, screening is performed in SNDS to identify new cases to be included in the cancer cohort, and to retrieve healthcare consumption data of subjects newly and already included.

### Data sources

The cancer cohort is an extract from SNDS based on health service use and reimbursement. It contains individual data used for the billing and reimbursement of outpatient healthcare consumption collected in the consumption database of the various national health insurance schemes (*Données de Consommation Inter-Régimes* database, DCIR) and private and public hospital database, collected in the medical information system programme *(Programme de Médicalisation des Systèmes d’Information*, PMSI) by the agency for information on hospital care (ATIH) [10]. As in the SNDS, subjects included in the cohort are identified by an anonymised personal identifier, based on the social security number.

DCIR contains personal and social characteristic data such as date of birth and death (if applicable), gender, post code, information on benefits indicating specific reimbursements for low income status such as the *complementary universal health insurance (*i.e. an access to social protection for all French or foreigners stably and regularly living in France for more than three months, with or without a fixed address and with a low income status). Medical characteristics are also recorded such as the presence of costly or long-term illnesses (LTIs), entitled to 100% reimbursement of expenditure. Cancer is an LTI, the diagnosis of which is encoded according to the International Statistical Classification of Diseases and Related Health Problems - 10th Revision (ICD-10). Moreover, outpatient healthcare consumption and costs are recorded, such as: date and nature of medical consultation and paramedical intervention, medication prescription and delivery with dates, list of specific products or medical devices reimbursed, medical procedures, lab tests (but without the results), medical transport, occupational accidents and illnesses, and disability. Data are entered or recorded according to various specific guidelines and national or international classifications [10].

Furthermore, for patients affiliated to the General schemes, 56 groups of chronic diseases, health events, or chronic treatments (combined in 13 main categories), and the ecological deprivation index ('FDep99’) are identified [11, 12].

PMSI, which is based on diagnosis-related groups (DRG), describes hospital stays and costs in conventional medical units (short stays—MCO), follow-up rehab care units (SSR), homecare units (HAD) and psychiatric units. These databases all contain demographic (age, sex, town/city, post/zip code, vital status at the end of the stay) and medical information including diagnoses and medical procedures using the same classification as in DCIR. They also contain consumption of expensive drugs and devices, not included in the DRG pricing.

### Inclusion criteria

**Fig 1:**
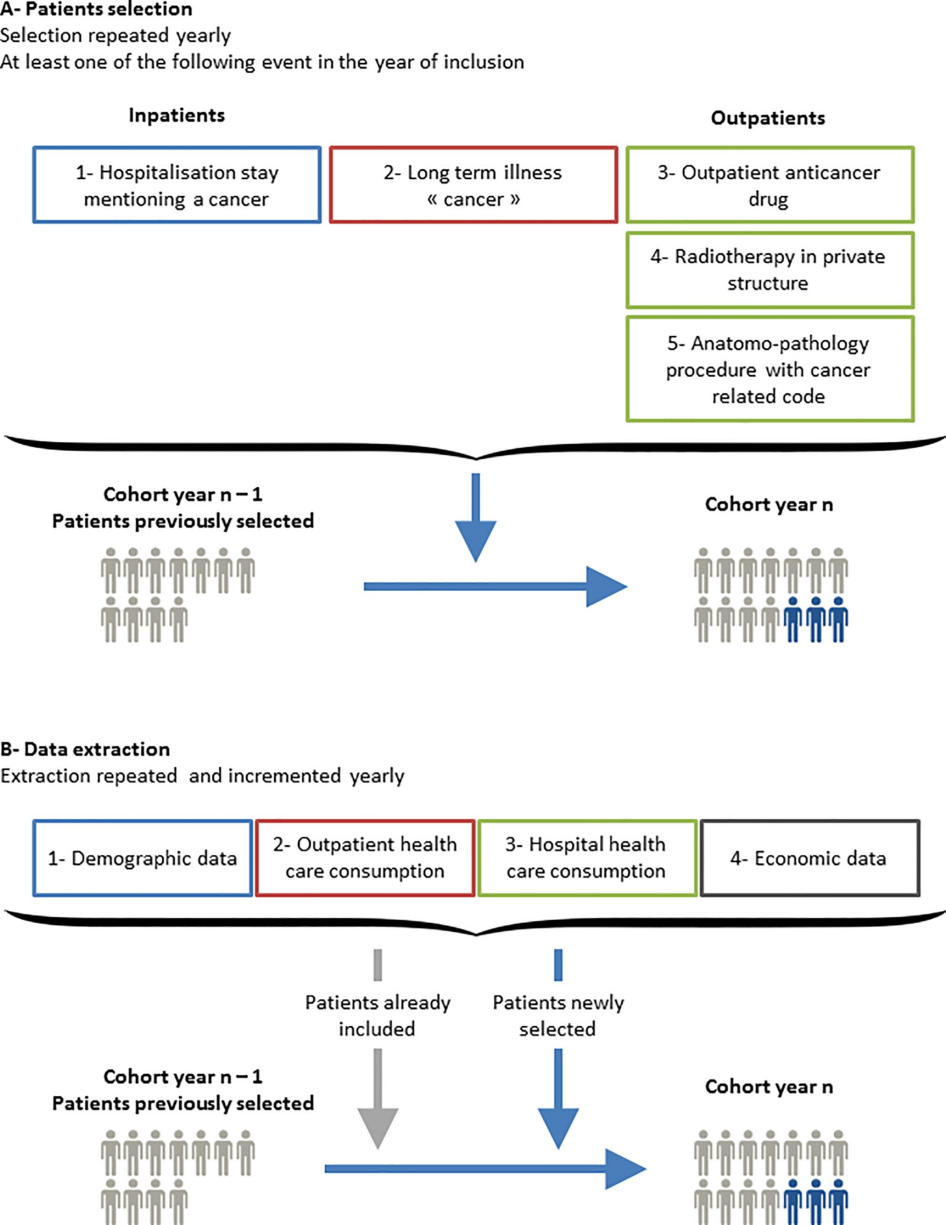
Patient selection and data extraction.

Five conditions are considered for inclusion. Subjects are included in the cancer cohort if they had at least one of these following events in the year of inclusion: 1) They had a hospital stay recorded in the PMSI MCO database and flagged by the cancer algorithm developed by INCa in collaboration with French Hospital Federations and ATIH [13]. This concerns patients hospitalised for cancer diagnosis, those receiving curative, palliative or preventive treatment (persons at high risk of cancer); those monitored during or post-cancer treatment; and those hospitalised for the management of a complication or the consequences of cancer and treatment side-effects. 2) They were registered as having LTI status due to their cancer. 3) They received an outpatient anticancer drug. 4) They received radiotherapy in a private structure (radiotherapy given in public structure is already included in PMSI MCO). 5) They had an anatomopathology procedure with a cancer-related code. The first condition was derived from the PMSI database, and the last four, from the DCIR database (Fig 1).

### Data extraction and update

The cohort is updated once a year by the French Health Insurance. New cases to be included in the cancer cohort are identified every year. Healthcare consumption is extracted every year for all patients (those already included and those newly included) and the vital status is updated (Fig 1).

### Data check and exclusion criteria

INCa checks new sets of extracted data in order to verify consistency, such as the volume of data, the presence of duplicates, the rate of hospital stays identified by the cancer algorithm and actually included in the cancer cohort, the rate of cancer-related healthcare consumption, *etc*. Rules are applied to compensate for some discrepancies, leading to exclusion, mainly: a) subjects included via the LTI source deceased prior to their entry into the cohort (because the LTI end date was not systematically updated after death, information type 2); b) subjects only receiving an outpatient anticancer drug which also has an indication other than cancer because we supposed that they are not treated for cancer (for instance, someone who received methotrexate but had no LTI, no hospitalization, no radiotherapy or anatomopathology procedure for cancer was probably treated for an inflammatory disease, information type 3); c) subjects having only undergone a procedure by a radiotherapist in a private structure which is not radiotherapy-related (information type 4).

### Data type [10]

The cohort includes 1) demographic data such as gender, age (birth data), rank of birth (for multiple pregnancies), geographical code of the town of residence, date and cause of death and health insurance coverage. 2) The nature and date of procedures performed by general practitioners and specialists, dentists, midwives, physiotherapists, speech therapists, orthoptists, nurses and podiatrists-chiropodists, in their offices, at the patient's home, in private clinics or in certain health or medical and social welfare centres are recorded. 3) Hospital health care consumption corresponds to hospitalisations in short-stay institutions, aftercare and rehabilitation, psychiatry and hospital at home. The available information is derived from the anonymous discharge summaries established at the end of each stay. 4) Economic data present concern office care, stays in private institutions, medical and social welfare sector expenditure. For hospital stays and outpatient consultations and procedures in public institutions, economic data are available in stay valorisation tables.

### Committees

Two committees have been set up in order to follow up the cancer cohort project and provide a scientific and ethical board.

The steering committee participates in the development of work in order to analyse data and make results available through reports and performance indicators. It issues technical advisory opinions on methods and analyses. It is composed of experts and representatives from the four national hospital federations and various French health agencies.

The scientific advisory board suggests guidelines concerning work streams, potential development, and collaborations. It follows up projects and validates results, facilitates exchanges between the various parties involved and helps develop methods and results. Furthermore, it reviews the suitability of requests for access to the cancer cohort (including scientific and ethical considerations). It is composed of delegates from the four national hospital federations, various French health agencies, the main three compulsory health insurance schemes, and experts.

### Ethics and data protection

The French cancer cohort protocol was approved by a national committee (*Comité Consultatif sur le Traitement de l’Information en Matière de Recherche dans le Domaine de la Santé*, study registered under n°22/2011), and authorized by the French data protection Agency (*Commission nationale de l’informatique et des libertés*—Cnil, study registered under n°911297). Confidentiality is guaranteed for all participants with regard to any personal information, as all data are pseudonymised.

### How to access the cancer cohort

Scientific protocols must be submitted to the French national cancer institute and reviewed by national committees Cerees (*Comité d’expertise pour les recherches*, *études et évaluations en santé*) and Cnil. Prior submitting a protocol, a contact is mandatory (lesdonnees@institutcancer.fr). We develop documentation and propose a specific support in order to better characterise information and analyses plans.

## Short description of the cohort database

### Number of subjects included since 2010

**Fig 2:**
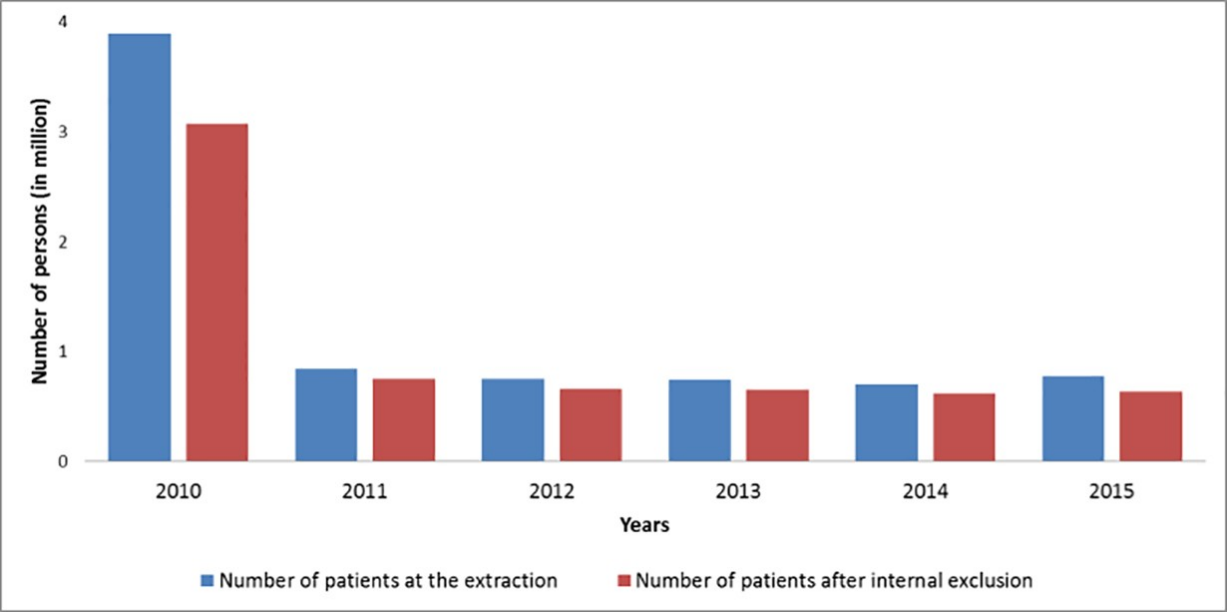
Number of patients included in the cancer cohort between 2010 and 2015.

Since 2010, 6.4 million subjects have been included in the cancer cohort after internal exclusions, among the 7.7 million with at least one information type (Fig 2). Most subjects were entered in the cohort in 2010 (3 million after exclusion), including incident and prevalent cases. The number of subjects included has decreased since then, from 0.7 million in 2011 to 0.6 million in 2015, and is trending towards the incident population (including all types of diagnosed cancer: invasive, in situ and tumour with uncertain behavior).

### Information type for inclusion (before exclusion)

**Fig 3:**
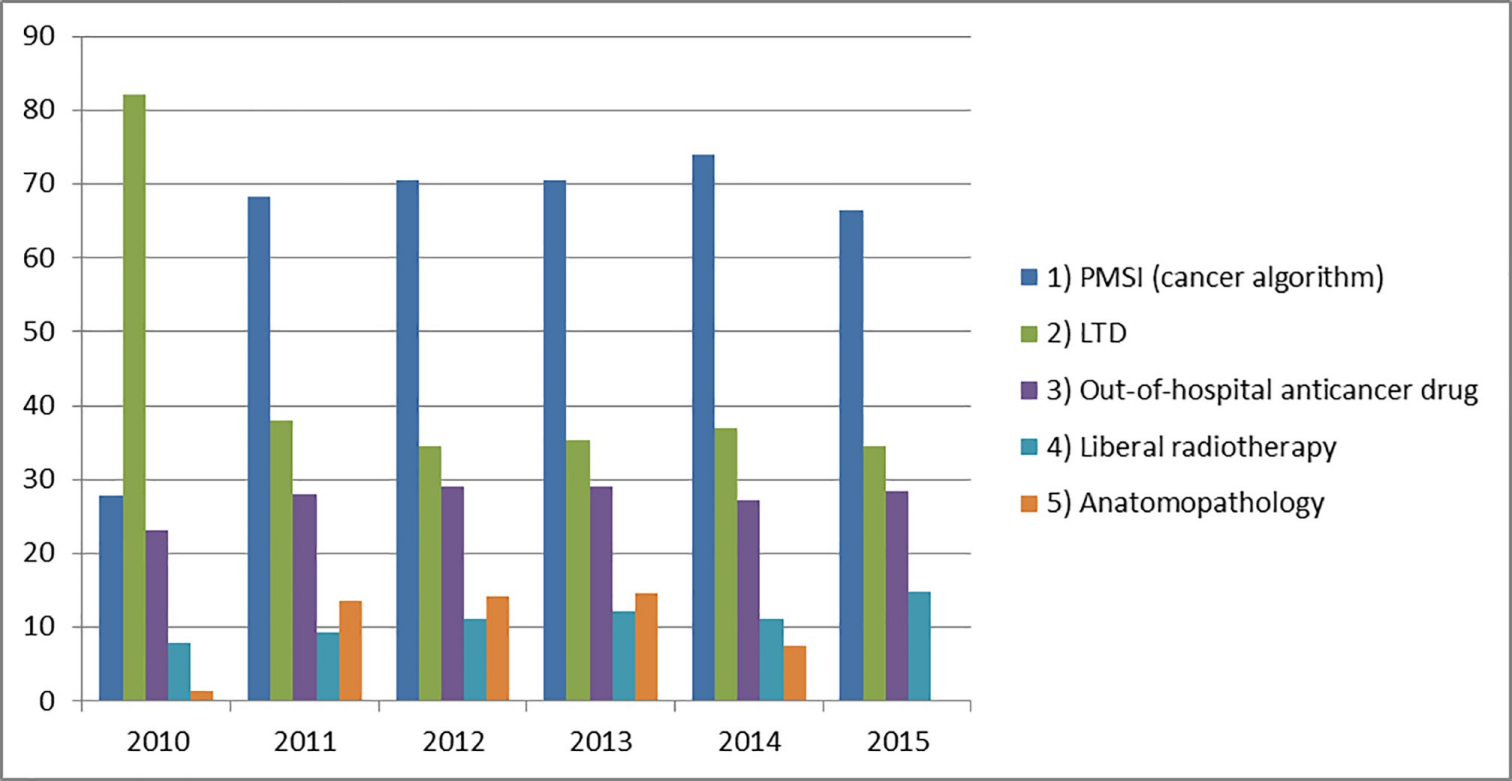
Information types for inclusion by subject between 2010 and 2015.

In 2010, the most frequent information type for inclusion was LTI with 82% of subjects included via this source. Since 2011, hospital stays identified by the cancer algorithm represented the first information type for inclusion, followed by LTI. In 2015, 67% and 35% of subjects respectively were concerned by these information types, given that subjects could be included via several information types (Fig 3).

### Exclusion

In 2010, more than 700,000 subjects (18%) died before the implementation of cohort data collection, 99.9% of whom because the LTI end date had not been updated after death. Since 2011, less than 0.001% of subjects have been excluded for this reason; entry in the cohort with only outpatient anticancer drug consumption (information type 3) being the main reason for exclusion.

### Subject characteristics

Subjects included in the cohort (and not excluded during data management) had a median age of 69 [58–78] years in 2010 and 65 [54–76] in 2015; 1% had less than 18 years (Table 1). In 2010 and 2015, around 51% were women and 84% were insured under the General scheme (the major health insurance scheme covering salaried employees of the private sector and their dependents). In 2010, and 84% had a registered LTI (47% in 2015). In 2015, 40% of subjects included underwent surgery for cancer, 16% chemotherapy at hospital and 11% at least one session of radiotherapy.

**Table 1:** Characteristics of subjects benefiting from cancer-related healthcare in 2010 and 2015.

Year of inclusion in the cohort		2010	2015
Number of persons included (and not excluded)		3 142 010	578 384
Age (median)		69	65
Age (%)	< 18	1	1
	18–50	11	16
	50–74	51	55
	> 75	35	28
	Not available	2	0
Gender (%)	Men	48	49
	Women	51	51
	Not available	2	0
Health insurance scheme (%)	General scheme (including local mutualist sections)	84	84
	Scheme for agricultural workers	4	6
	Scheme for self-employed workers	7	6
	Others schemes	3	4
	Not available	2	0
Deaths (%)		6	9
Complementary universal health insurance (for <60 years) (%)		2	4
LTD for cancer (%)		84	47
Surgery for cancer (%)		12	40
Chemotherapy at hospital (%)		9	16
Radiotherapy (%)		6	11
Most frequent cancer localisation (%)	Breast	24	13
	Prostate	17	10
	Colorectal	12	16
	Lymphoid and hematopoietic tissue	11	11
	Skin	6	13
	Lung	6	8

In 2015, the primary cancer organ could be attributed to 96% of the people (excluding those who had a surveillance for a family history), using ICD-10 codes. The most frequent locations were colon-rectum (16%), skin (14%), breast (13%), lymphoid and hematopoietic tissue (11%), prostate (10%) and lung (8%) (Table 1). Finally, 19% had a tumour with uncertain behavior. People without primary organ identifyed were mainly included via an isolated out-of-hospital anticancer drug (51%) or a hospitalization related to cancer without a cancer code relative to a specific organ (37%).

### Big data impact

The cancer cohort represents several terabytes of data, including around 80 tables for PMSI each year, 10 for SNIIRAM, and 250 tables of values or data repositories. In addition, INCa’s data manager in charge of the cohort creates or updates home repositories. To date, the entire row database represents more than 14 billion records distributed in nearly 400 tables.

## Some studies as a case in point

The cancer cohort allows disease-based longitudinal studies on care pathways. For instance, a study on breast cancer focused on surgery (partial and total mastectomy), chemotherapy and radiotherapy according to the breast cancer stage (in situ, local and regional) is ongoing, based on previous research [14]. This research pointed out that the intervals between surgery and chemotherapy were in keeping with the guidelines for 98% of the women. However, the interval between chemotherapy and radiotherapy was longer than recommended for 40% of the women. This research is being repeated in order to observe potential trends, and extended to the diagnostic phase (including breast cancer mass screening) and outpatient care.

Some studies relative to drug utilisation, drug effectiveness or adverse effects have been launched. One of them focuses on anti-PD-1 and anti-PD-L1 (programmed death-1 and programmed death ligand-1).

The aim of this study is to analyse data that cannot be obtained, or only obtained in part, from randomised clinical trials. This real-world follow-up includes the number of treated patients, in accordance with the marketing authorisation or off-label use, dose and duration of anti-PD-1 treatment, previous treatments, combination with other therapy, permanent or temporary discontinuation.

The cohort is also used to evaluate healthcare and public health policies. For instance, in the field of fertility preservation, as outlined in a report co-produced by the French National Cancer Institute and the French Biomedicine Agency on the consequences of cancer treatments on reproduction and preservation of fertility [15], access to fertility preservation is currently not available for all the subjects concerned. A better approach in daily practice is mandatory. Medical professionals in oncology but also in fertility preservation have to better integrate in their daily practice fertility preservation in cancer patients. By consequence, a study based on the cancer cohort was conducted to define the target fertility preservation number required at national and regional level [16]. Each year, between 17,200 and 40,000 cancer patients of reproductive age according to the limit age should be informed about the risks to their future fertility of the treatments offered and about the fertility preservation options available.

## Discussion

Based on medico-administrative data from SNDS, the cancer cohort has been designed to study cancer care in the short-, medium- and long-term after primary tumour diagnosis, and evaluate public health policies for cancer, in order to help policymakers. With more than 7 million subjects included between 2010 and 2015, the cancer cohort is one of the largest cancer databases in the world. Each year, new cancer cases will be included and followed up for 25 years. One of the major strengths of the cohort is that it covers the entire French population (i.e. more than 66 million inhabitants) regardless of national insurance system or socioeconomic status.

The development of such a cohort was a long process due to of the many pitfalls to be resolved. One year after discussing the need to have a big data cohort to observe cancer care in real life, the decision to create the cancer cohort was taken in 2010. Indeed, with this cohort, persons could be followed for 25 years which will allow evaluating second cancers and survival. Moreover linkage to other cohorts is feasible. The Cnil authorized the project in 2012. In 2013–2014, work was initiated in connection with French Health Insurance which is the body in charge of French medico-administrative data, and the National health data institute which helps users to access data. The first step consisted of identifying subjects to be included in the cohort. INCa received a first set of data in 2014, worked on the validation of the extraction, and finally received the complete set for 2010–2014 in 2015. Data for 2015 were obtained in March 2017. For each extraction, a validation process was defined to check data consistency, to be sure that the data could be analysed. Moreover, as a consequence of the reimbursement claim that could occurred within two years after the medical consumption, 700 000 persons were excluded from the cohort. Thus, these persons deceased prior to 2010 but remained in the SNDS database for two years after their death. At the present time, the cohort is routinely updated once a year.

In routine use, the cancer cohort can also be used to produce performance indicators. First indicators developed will concern healthcare activity (radiotherapy, outpatient and hospital medical consumption, cancer-related surgery, chemotherapy or palliative care). Performance indicators focusing on specific populations (such as paediatric or geriatric populations) will differ from those for the general population.

The cohort is a major tool for epidemiological purposes. Its advantages are its exhaustiveness, prospective data collection and the speed of implementation. One of the weaknesses is a lack of clinical data from medical records. However, the cohort is based on medicalised databases, and we can construct algorithms to approach these clinical data. In most cases, identification of disease requires a combination of different ICD-10 codes from LTI and hospital diagnoses, and disease-specific drugs or procedures. Furthermore, although biological results or conclusions of medical procedures are lacking, the knowledge of biological test performances and medical procedures is informative, and strong assumptions could be proposed in regards to specific medical procedures, repetition of examinations, or use of specific treatments. Consequently, the construction of algorithms to identify clinical data requires a multidisciplinary approach involving epidemiologists, statisticians, medical information department physicians, clinicians or national health insurance personnel. To date, several studies have been published in the field of cancer using such algorithms on SNDS [17–22, 14, 8, 23, 24] [25]. For example, French Health Insurance has developed a disease and expenditure mapping tool, using such algorithms, which was previously used to describe the human and economic burden of cancer in France in 2014 [11, 8]. This tool will allow identifying easily comorbidities for patients affiliated to the General scheme. Unfortunately, at the present time, it is not easy to evaluate these algorithms with systematic referencing to medical files or medical data collected by French cancer registries. Validation studies are scarce concerning cancer algorithms developed on medico-administrative databases, often with probabilistic matching [26–31]. Without external validation, it would appear to be important to describe the algorithms used precisely and to perform sensitivity analyses.

Otherwise, other important information is not available in the cancer cohort such as the TNM (Tumour, Nodes and Metastasis) cancer classification, and must be addressed using ICD-10 codes [14]. Non-reimbursable drugs, self-medication, and drugs dispensed during hospital stays (except expensive drugs not included in DRG pricing) are not included in SNDS, which limits the study of medication protocols.

In the future, nationally standardised data from cancer medical records could be linked to the cancer cohort, allowing us to obtain information on cancer histopathology, stage of cancer, or exact medication protocols for example, and easily validating algorithms developed on SNDS in relation to cancer [22]. Since end of 2017, death certificates have been included in the SNDS; they will be extracted for the cohort cancer in 2018. Moreover, in the future, SNDS (and by extension the cancer cohort) will include data from the National old age pension fund for employees recording individual working careers. Professional pathways will be set up and linked with both cancer causes and impact on socioeconomic life.

This type of linking, between claims data and clinical data, has already been conducted abroad [26, 27, 29, 31], such as in the USA with the SEER-Medicare database, which provides detailed information on cancer site, stage, and histology that is not available in Medicare claims data. This database can be used for epidemiological and health service research, such as for studies on patterns of care for subjects with cancer, on the use of cancer tests and procedures or on the costs of cancer treatment [32].

One of the main pitfalls is inherent to the database, with its vast amount of information, and the complexity of its structure. It requires technical experience and knowledge on the large amount of tables and variables. In order to facilitate use, we have created simplified tables with aggregated information. Moreover, the cancer cohort is part of the new large databases in the big data field. New technologies that could markedly modify the database and its development are reviewed, such as NoSQL procedures.

The cancer cohort offers massive potential for studies in the field of cancer. These studies will be: cross-disciplinary, on cancer care and costs, and will result in performance indicators; longitudinal, dealing with care pathways, risk of second cancer, medium- and long-term effects of anticancer drugs, trends in the costs of cancer care, medium- and long-term impact of the introduction of public cancer policies or guidelines for health professionals. In order to exploit this potential, collaborative studies have been initiated with several research teams.
